# Prostate Cancer—Focus on Cholesterol

**DOI:** 10.3390/cancers13184696

**Published:** 2021-09-19

**Authors:** Lucija Škara, Ana Huđek Turković, Ivan Pezelj, Alen Vrtarić, Nino Sinčić, Božo Krušlin, Monika Ulamec

**Affiliations:** 1 Department of Medical Biology, School of Medicine, University of Zagreb, 10000 Zagreb, Croatia; nino.sincic@mef.hr; 2 Group for Research on Epigenetic Biomarkers (Epimark), School of Medicine, University of Zagreb, 10000 Zagreb, Croatia; monika.ulamec@kbcsm.hr; 3 Centre of Excellence for Reproductive and Regenerative Medicine, School of Medicine, University of Zagreb, 10000 Zagreb, Croatia; bozo.kruslin@kbcsm.hr; 4 Faculty of Food Technology and Biotechnology, University of Zagreb, 10000 Zagreb, Croatia; ana.hudek@pbf.unizg.hr; 5 Department of Urology, University Clinical Hospital Center Sestre Milosrdnice, 10000 Zagreb, Croatia; ivan.pezelj@kbcsm.hr; 6 Department of Clinical Chemistry, University Clinical Hospital Center Sestre Milosrdnice, 10000 Zagreb, Croatia; alen.vrtaric@kbcsm.hr; 7 Ljudevit Jurak Clinical Department of Pathology and Cytology, Sestre Milosrdnice University Hospital Center, 10000 Zagreb, Croatia; 8 Department of Pathology, School of Medicine, University of Zagreb, 10000 Zagreb, Croatia

**Keywords:** prostate cancer, cholesterol, SREBP2, PTEN, mTOR: MAP, p53, AR

## Abstract

**Simple Summary:**

Prostate cancer presents a significant global public health burden. One of its established risk factors is high fat diet. It has been proven that cholesterol levels in blood and prostate tissue are out of balance, while cholesterol metabolism in prostate cancer is deregulated and plays an important role in cancer progression. In this review we have shown the connection between commonly deregulated pathways in prostate cancer and cholesterol metabolism.

**Abstract:**

Prostate cancer (PC) is the most common malignancy in men. Common characteristic involved in PC pathogenesis are disturbed lipid metabolism and abnormal cholesterol accumulation. Cholesterol can be further utilized for membrane or hormone synthesis while cholesterol biosynthesis intermediates are important for oncogene membrane anchoring, nucleotide synthesis and mitochondrial electron transport. Since cholesterol and its biosynthesis intermediates influence numerous cellular processes, in this review we have described cholesterol homeostasis in a normal cell. Additionally, we have illustrated how commonly deregulated signaling pathways in PC (PI3K/AKT/MTOR, MAPK, AR and p53) are linked with cholesterol homeostasis regulation.

## 1. Introduction

Prostate cancer (PC) with high incidence (>1.1 million new cases each year) and mortality (~300,000 deaths per year) presents a significant global public health burden [1,2]. Established risk factors of PC are age, race and family history of disease [3,4]. Although, many risk factors are non-modifiable, association with preventable high fat diets, risk of PC and progression of the disease have been observed in different studies [5,6,7,8,9]. High fat diet increases total and low-density lipoprotein (LDL) cholesterol and decreases high-density lipoprotein (HDL) cholesterol in plasma [10]. Experiments on cell culture, xenografts, clinical samples and epidemiological studies confirm aberrant cholesterol metabolism in PC and its importance in progression [11,12,13].

In this review, we summarized current knowledge related to cholesterol metabolism and its function in normal cells. Furthermore, we illustrated how commonly deregulated signaling pathways in PC reflect cholesterol metabolism. 

## 2. Prostate Metabolism

The prostate is a gland in the male reproductive system which has the main purpose of secreting prostatic fluid which supports spermatozoa during fertilization. Prostate exhibits a unique metabolism adapted to meet the demands of its function; production of prostatic fluid, mostly consisting of citrate, zinc, kallikrein enzymes and cholesterol [14,15,16]. ATP production in normal prostate cells, especially epithelial cells of the prostate peripheral zone, rely on glycolysis. Acetyl-CoA generated from glucose and oxaloacetate generated from aspartate are used for citrate synthesis. Citrate is not intended for oxidation but for secretion, which is achieved by importing and accumulating a high concentration of zinc, a unique characteristic of prostate cells which inhibit m-aconitase, an enzyme for citrate-isocitrate conversion, stopping the Krebs cycle with citrate as end-product [17,18]. Along with higher glycolytic activity and reduced oxidative phosphorylation, normal prostate epithelium has higher cholesterol synthesis than other tissue types and further increases with aging [10], while fatty acids (FAs) are mostly diet-derived [19]. Metabolic reprogramming is a characteristic of cancer cells [20]. Cancer epithelial cells of the prostate peripheral zone have lower zinc concentration (70–80%) than normal counterparts, with no exception. Decline in zinc and citrate is an early occurrence in malignant transformation, while the protein level of aconitase is maintained [17,20]. This results in decrease of citrate in semen and a metabolic shift to citrate oxidation and oxidative phosphorylation in primary PC, thus the early stage of PC does not exhibit the Warburg effect [21]. Citrate exported in cytoplasm is now cleaved to acetyl-CoA by enzyme ATP citrate lyase (ACLY) and further used for de novo FA synthesis. Synthesis and uptake of FA and cholesterol accumulation in PC are increased regardless of blood lipid levels [14,19,22]. Primary PC relies on oxidation of de novo synthesized or exogenous FA, so anabolism and catabolism of FA co-occur in the same cell [14]. Since glucose uptake in primary PC is modest, its imaging by 18F- fluorodeoxyglucose-positron emission tomography (FDG-PET) is limited. In that case, labeled acetate or choline are more useful tracer molecules due to increased acetate uptake and choline kinase up-regulation [14,23]. Advanced PC becomes glycolytic again, so FDG-PET could be effective in the advanced stages of PC and biochemical recurrence, prediction while at the same time has increased FA synthesis [15,21,23].

## 3. Cholesterol Function and Prostate Cell Supply

Cholesterol accounts for approximately one third of lipids in plasma membrane [24]. Amphiphilic and almost planar molecules of cholesterol are important components of the cell membrane, regulating its integrity, fluidity, and permeability [25]. Cholesterol is essential for cell cycle progression and differentiation, while cholesterol depleted cells are arrested [26]. Cholesterol-enriched micro domains, lipid rafts, serve in signal transduction as platforms for recruiting receptors and their downstream targets. Many important regulators of cell growth, cell adhesion, migration and apoptosis are located in lipid rafts, such as epidermal growth factor receptor (EGFR), mitogen-activated protein kinase (MAPK), Src family kinases, protein kinase C, caveolins, flotillins and others [27,28]. Cholesterol is important for cancer stem cell maintenance, it covalently bounded to smoothened receptors and activates the oncogenic Hedgehog signaling [29,30,31]. In addition to its role as a fundamental structural component of the cell membrane, cholesterol is a precursor of oxysterols, bile acids, steroid hormones, and vitamin D [32]. Cellular cholesterol could either be imported from extracellular source as lipoprotein or endogenously synthesized de novo through the mevalonate pathway [25]. Cholesterol in blood originates from either the diet or liver metabolism [33]. The majority of exogenous cholesterol is transported in LDL particles which binds to LDL receptor (LDLR) [31]. This complex receptor-ligand is then internalized and delivered into early endosomes via clathrin-mediated endocytosis. As endosome acidifies, the LDL receptor dissociates from the complex and returns to the plasma membrane while an LDL particle remains in the maturing endosome/lysosome and is attacked by the lysosomal acid lipase type A that hydrolyzes cholesteryl ester to release free cholesterol. One in 500–1000 LDL particles is associated with plasma proprotein convertase subtilisin/kexin type 9), which directs LDLR to lysosome degradation, thus reducing LDLR membrane density and leading to an increase in plasma LDL [34]. Excessive intracellular cholesterol is transferred by the ATP-binding cassette transporter A1 (ABCA1) and the ATP-binding cassette transporter G (ABCG1) to high-density lipoprotein (HDL) particles. HDL particles return excessive intracellular cholesterol to the liver or intestine where it is recycled, excreted or delivered to the steroidogenic organs and utilized for hormone synthesis [35]. The multiligand membrane receptor protein scavenger receptor class B member 1 (SR-B1) binds to the HDL particle and facilitates cholesterol efflux from the cell, but also can facilitate cholesterol influx [36,37]. De novo cholesterol synthesis occurs in the cytoplasm and includes about 30 subsequent reactions. Starting molecule is acetyl-CoA which can be obtained from citrate by cytoplasmic ACLY, acetate by acetyl-CoA synthetases and from pyruvate by pyruvate dehydrogenase [38,39]. Cholesterol synthesis is a complex and energetically expensive process which begins with condensation of two acetyl-CoA molecules into aceto-acetyl-CoA by cytosolic enzyme acetyl-CoA acetyltransferase 2. [40] Aceto-acetyl-CoA can also be derived from acetoacetate produced during keto-genesis is by mitochondrial enzyme aceto-acetyl-CoA synthetase 1. Condensation of the third acetate molecule with aceto-acetyl-CoA leads to 3-hydroxy-3methylglutaryl-CoA (HMG-CoA) production. 3-hydroxy-3-methylglutaryl-CoA reductase (HMGCR) catalyzes the HMG-CoA reduction to mevalonate, and this is the first rate-limiting step of cholesterol synthesis. HMGCR is regulated via a negative feedback mechanism. Higher cholesterol and 25-hydroxycholesterol concentrations decrease HMGCR synthesis and accelerate its degradation. Rate-limiting steps are catalyzed by HMGCR and squalene monooxygenase (SQLE). An increase in the concentration of cholesterol or 25-hydroxycholesterol suppresses the synthesis of HMGCR and leads to a marked decrease in HMGCR; furthermore, cholesterol accelerates HMGCR degradation by facilitating HMGCR ubiquitination. These two mechanisms result in a synergistic effect, ultimately leading to a decrease in both the HMGCR concentration and cholesterol biosynthesis to decrease the concentration of cholesterol. Through several consecutive reactions mevalonate is converted into squalene [41,42]. Synthesis of squalene is the second rate limiting step and the first cholesterol specific step. 

Besides sterol intermediates, non-sterol intermediates are produced [35]. The mevalonate pathway is also the source of ubiquinone, dolichols, hem, isopentenyl-diphosphate, farnesyl, and geranyl groups. Although cholesterol plays a significant role in the cell, high levels of it are toxic. To prevent free cholesterol toxicity, excessive free cholesterol is esterified by acyl-Co acyltransferases and stored in cytoplasmic lipid droplets together with neutral lipids and coat proteins or is exported from cells via ABCA1 and ABCG1 [43]. Cellular cholesterol homeostasis is maintained within physiological range by complex interplay among synthesis, uptake, efflux, and esterification [44].

## 4. Regulation of Cholesterol Homeostasis

**Figure 1:**
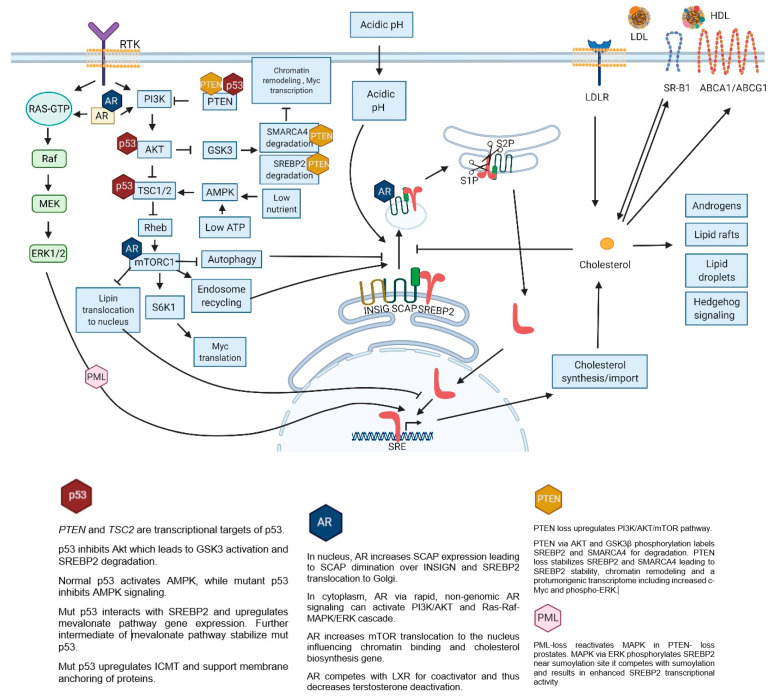
SREBP2 activation and regulation in physiologically normal cell. Hexagons indicate points of influence by commonly aberrant pathways in PC. Receptor tyrosine kinase (RTK); androgen receptor (AR); glycogen synthase kinase 3 (GSK3), high-density lipoprotein (HDL); low-density lipoprotein (LDL); low-density lipoprotein receptor (LDLR); ATP-binding cassette transporter A1 (ABCA1); ATP-binding cassette transporter G1 (ABCG1); scavenger receptor class B member 1 (SR-B1); insulin-induced gene (INSIG); SREBP cleavage-activating protein (SCAP); site-1 protease (S1P); site-2 protease (S2P); sterol regulatory element–binding protein-2 (SREBP2); sterol response element (SRE). Created with BioRender.com (accessed on 5 September 2021).

Depending on the quantity of absorbed cholesterol, cholesterol synthesis and excretion will be adjusted accordingly. Absorbed cholesterol is derived from food, bile and shedding of intestinal epithelium. The main site of cholesterol absorption and secretion is the intestine, while the liver is the main site of cholesterol synthesis, but almost all cells can produce cholesterol [45,46]. Cellular cholesterol balance is maintained by two main transcription factors: sterol regulatory element–binding protein-2 (SREBP2) and liver X receptors (LXRs) [25]. SREBPs are transcription factors that regulate gene expression included in lipid synthesis and function with global biological signaling pathways involved in various physiological and pathophysiological processes. The SREBP family of transcription factors consists of two genes coding for three proteins. Isoforms SREBP1a and SREBP1c are coded by SREBF1 gene and are more efficient in transcriptional regulation of genes involved in fatty acid synthesis, while SREBP2 regulates genes involved in cholesterol synthesis [47]. SREBP2 is synthetized on the endoplasmic reticulum (ER) membrane as inactive 125 kDa precursor (Figure 1) [48]. In order to avoid degradation, it has to be in constant contact with SREBP cleavage-activating protein (SCAP) which binds SREBP2 immediately after SCAP synthesis [49,50]. SCAP poses a sterol-sensing domain and coatomer II (COPII) export signal. When cholesterol in a cell is high, it binds to SCAP triggering conformational changes that separate loop 1 from loop 7. Conformational changes now allow interaction between SCAP and anchor proteins insulin-induced gene (INSIG) proteins. When associated with SCAP, INSIG1 is stabile, COPII export signal is masked and consequently SCAP/SREBP2 complex remains trapped in the membrane [50].

When cholesterol in ER drops under ∼5%, or under 3% and when INSIG1 is overexpressed, loop 1 and loop 7 of SCAP interact. This loops interaction is probably secured by polyubiquitination of SCAP mediated by ring finger protein 5. COPII export signal is exposed, SCAP binds COPII and SCAP/SREBP2 complex is transported to Golgi [51]. Soon after, SCAP/SREBP2 dissociation forms INSIG1 which is ubiquitylated by E3 ubiquitin ligase GP78 and marked for proteasomal degradation [50,52]. GP78 when attached to INSIG also ubiquitinates HMGCR [53]. Heat shock protein 90 stabilizes SREBP2/SCAP complex all the way from ER to the Golgi and in the Golgi [35]. SREBP2 is proteolytically activated in the Golgi by Site-1 protease (S1P) and Site-2 protease (S2P). Cleaved SREBP2 enters the nucleus and, with the help of chromatin remodeling protein SMARCA4, binds to the sterol response element (SRE) sequence. SREs are located in promoters of genes involved in cholesterol biosynthesis, import, and also in INSIG1 gene. INSIG2 gene is not under SREBP2 control but under insulin [35,51,54,55,56].

The activity of cleaved SREBP2 in the nucleus is further tuned by phosphorylation, ubiquitination, and sumoylation. Phosphorylation of nuclear SREBP2 by AMPK attenuates while phosphorylation by extracellular signal-regulated kinase (ERK) enhances SREBP2 transcriptional activity [35]. Cleaved SREBP2 in the nucleus is directed to proteasome by Fbw7-mediated ubiquitination, a process that depends on prior glycogen synthase kinase 3 (GSK3) phosphorylation [35,57]. SREBP2 protein stability is enhanced by transcriptional coactivator p300 acetylation [58]. SREBP2 sumoylation reduces transcriptional activity since it induces recruitment of a corepressor complex containing HDAC3. Since MAPKs phosphorylate SREBP2 near the sumoylation site, it competes with sumoylation and results in reversed SUMO-induced SREBP2 transcriptional activity attenuation [59]. NAD+-dependent protein deacetylase SIRT6 is the only sirtuin that directly regulates the mammalian lifespan. By deacetylating H3K56 in the promoter, SIRT6 acts as negative regulator of SREBP2 transcription while, by decreasing protein levels of S1P, S2P and SCAP, it suppresses SREBP2 cleavage [60].

Oxysterols are cholesterol molecules that have been oxidized either by a specific enzyme or by reactive oxygen species (ROS) [61]. Similarly, cholesterol, which blocks SREBP2 processing by binding to SCAP and consequently SCAP anchoring via INSIG, oxysterols (such as 24-, 25-, and 27-hydroxycholesterol) block SREBP2 processing by binding to INSIG which than binds to SCAP [41,62]. Therefore, INSIGs are described as oxysterol-binding proteins. Oxysterols are ligands for the LXR nuclear receptor. Isoform LXRα is coded by NR1H3 gene and expressed in some tissues, while LXRβ isoform is coded by NR1H2 gene and ubiquitously expressed. LXRs forms heterodimers with retinoid X receptors and recognize LXR response elements in DNA. In the absence of a ligand, heterodimer binds a co-repressor but binding of a ligand causes replacement of co-repressors with co-activators and transcription. LXR promotes transcription of genes involved in reverse cholesterol transport (ABCA1 and ABCG1), conversion to bile acids and MYLIP gene (also known as IDOL) leading to degradation of LDLR, very low density lipoprotein receptor and lipoprotein receptor-related proteins 8 (LRP8) [28,63,64]. LXR also directly suppresses expression of two cholesterologenic enzymes, CYP51A1 and FDFT1 [65].

In low energetic conditions, AMP-activated protein kinase (AMPK)phosphorylates and thus inactivates first rate-limiting enzyme, HMGCR. Second rate-limiting enzyme, SQLE, is degraded under high cholesterol conditions, but under high squalene (its substrate) conditions, degradation is prevented while enzyme activity is upregulated [66,67]. Normal prostate cells, because of their function, have higher cholesterol content. Prostate cell development and metabolism is significantly regulated by androgen receptor (AR). AR promotes cholesterol accumulation by upregulating SCAP, inhibiting LXR and downregulating genes for testosterone deactivation (described in Section 7.2) [68,69,70,71].

In summary, cholesterol homeostasis in the prostate cell is balanced by SREBP2 and LXR activity whose activity is modulated by ER cholesterol level, INSIG/SCAP ratio, transcriptional modifications, post-translational modifications, and mevalonate pathway metabolites and hormones. SREBP2 increases cholesterol synthesis, import and accumulation in response to low cellular cholesterol level. Increased cholesterol further suppresses SREBP2 activation and stimulates LXR which then decreases cellular cholesterol level by downregulating synthesis while promoting cholesterol efflux and LDLR degradation [53,69].

## 5. Cholesterol Profile in Blood

Cholesterol dysregulation is a common characteristic of PC and extensive lipid profiles of blood from PC, benign prostatic hyperplasia (BHP) and control patients have been made. Results are conflicting. Many studies have shown that cholesterol and LDL are significantly higher in PC and men with hypercholesterolemia are usually at higher risk of developing high-grade PC [72,73,74]. Moreover, one study suggests that extensive blood lipid profile could distinguish PC from BHP [73]. Some studies did not find a connection between LDL and overall PC risk but did find association of high HDL and increased risk of high-grade PC [75]. On the other hand, some studies did not find any connection between cholesterol, LDL or HDL and PC [24]. Possible causes of this discrepancy are prostate-specific antigen (PSA) PSA-driven biopsy, race difference and cholesterol uptake by PC or other tissue.

PSA positively correlates with total cholesterol and LDL among white men [76]. It is possible that PC is more frequently detected in patients with high cholesterol because, due to higher PSA, they are more frequently biopsied, but studies on cholesterol and PC connection did not take this into account [75]. PC overexpress LDLR but, due its small size, prostate probably cannot uptake enough cholesterol from blood to cause significant change, and that is why blood concentrations before and after prostate surgery do not change much [77]. Decrease in total cholesterol level as well as faster catabolism of LDL was recorded in patients with metastatic prostate cancer. It is not known whether clearance of LDL was done by prostate tissue or some other tissue, but this effect was already seen in hematological neoplasms [78,79,80]. Inter-individual genetic variability could contribute to confusing epidemiological results. 17 genomic locations associated with PC are also associated with LDL and triglycerides while there were no pleotropic loci shared between PC and HDL [81].

## 6. Cholesterol Profile in the Prostate Tissue

PC cells have higher concentrations of cholesterol in membrane, cytoplasm and are even two-fold in the nucleus when compared to normal counterpart [12,82]. Expression of *SREBP2* mRNA and protein is low in normal prostate tissue, higher in localized PC and the highest in metastatic castrate-resistant prostate cancer (CRPC). Its expression is associated with poor clinical outcomes [83]. As expected, genes involved in cholesterol synthesis and uptake are upregulated. *HMGCR* mRNA is higher in PC and is associated with earlier biochemical recurrence (BCR). High HMGCR protein expression in radical prostatectomy tissue is more frequent in tumor than in normal tissue and is associated with earlier BCR [84]. PC cells also have more lipid rafts, expressing more LDLR and cholesterol channel on the surface of mitochondria and nucleus peripheral-type benzodiazepine receptor than BPH and normal prostate cells [12,82]. Protein expression of cholesterol export gene *ABCA1* is lower in cancer prostate tissue and is negatively correlated with Gleason pattern [85]. SR-B1 is significantly more expressed in higher Gleason grade prostate tumors, and positively correlates with expression of enzymes involved in androgen synthesis which indicate role in intra-tumoral androgen synthesis and development of castration-resistant phenotype [86].

A study conducted on normal and different pathological human prostate tissues, cell lines and tumor xenografts reported accumulation of cholesteryl ester, dominantly cholesteryl oleate, in high-grade and metastatic human PC but not in normal prostate, prostatitis, BPH and prostatic intraepithelial neoplasia tissues. The study revealed that accumulated cholesterol in lipid droplets was not obtained from de novo synthesis, but rather by enhanced uptake of LDL [87]. On the other hand, numerous data point to de novo cholesterol synthesis prompting PC proliferation and progression [74]. PC bone metastases had significantly higher aggregates of cholesterol than metastases of other origins and normal bone. PC metastatic epithelial cells exhibited strong, homogenous LDLR staining, while staining of SR-B1 and HMGCR varied [88]. Uncontrolled LDLR and SREBP2 expression and cholesterol influx could be due to loss of sterol feedback regulation in prostate cancer cells [89].

## 7. Dysregulated Signaling Pathways in Prostate Cancer

Despite serum concentrations, cholesterol homeostasis in prostate cell is very tightly regulated and responds to cellular cholesterol and oxysterol levels and to extracellular stimuli by themechanisms described above. However, cholesterol level increases during progression to PC and accumulates in PC cells as a result of disturbed homeostasis in favor of cholesterolo-genesis [22]. Cholesterol dysregulation is involved in PC pathogenesis, besides being necessary for cell growth as membrane building block, and cholesterol contributes to intra-tumoral androgen synthesis [90]. Activation of different growth-promoting pathways is often connected with PC initiation while progression to CRPC primarily occurs with AR reactivation [91,92,93]. In contrast to other cancers, PC has relatively low overall mutation burden (0.3–2 non-synonymous somatic mutations per mega-base) [94]. Common genomic aberrations in prostate cancer are: TMPRSS2-ETS gene fusion, AR mutation and amplification, mutations and loss of tumor suppressors (TP53, PTEN, RB1, CHD1, BRCA2, APC, ATM), and mutations and amplifications of oncogenes (PIK3CA, MYC) [95]. In PC PIK3CA, BRAF, KRAS and AKT1, mutations are rare, but PIK3CA (catalytic subunit of PI3K) mRNA overexpression and gene amplification are frequent and correlate with pAKT and Gleason score, although a few cases indicate an additional mechanism involved in regulation of PIK3CA mRNA expression and pAKT [96,97].

The existing connection of altered genes and signaling pathways with SREBP2 and cholesterol will be described further (Figure 1). 

### 7.1. P53 and SREBP2

Up to 20% of PC harbor mutated *TP53*,frequently correlating with tumor grade, relapse and resistance to androgen [98]. Mutations usually occur within the DNA-binding domain eliciting loss of tumor suppressor and transcriptional abilities. Furthermore, mutant p53 can bind to wild type p53 and inactivate it [99]. p53 influences SREBP2 stability, activation, and transcriptional activity [100]. Mut p53 interacts with SREBP2 and upregulates mevalonate pathway gene expression. In turn, mevalonate-5-phosphate, intermediate in the mevalonate pathway, stabilizes misfolded mut p53 and protects it from a chaperone-dependent E3 ubiquitin ligase CHIP-mediated degradation, probably by enhancing mut p53-DNAJA1 interaction [99]. Mut p53 upregulates isoprenyl-cysteine carboxyl methyltransferase (ICMT) while mevalonate pathway generates farnesyl diphosphate (FPP) and geranyl-geranyl-pyrophosphate (GGPP) enabling oncogene prenylation, its docking to the cell membrane, and cancer progression [99,100].

p53, through other signaling pathways, indirectly influences SREBP2 destiny. Transcriptional targets of p53 are *PTEN* and *TSC2*, so p53 reduces activity of PI3K/Akt/mTORC1 signaling and SREBP2 translocation from ER, repressing transcription of mevalonate pathway genes and ICMT (methyl transferase acquired for protein prenylation) [101]. AMPK is kinase phosphorylated and activated in low nutrient or low energy levels. When activated it stimulates catabolism, suppresses SREBP2 cleavage, and by TSC2 protein inactivates the mechanistic target of rapamycin (mTOR). Wild-type p53 activates AMPK, while mutant p53 inhibits AMPK signaling and thus supports SREBP2 activation. The positive feedback loop between mut p53 and mevalonate pathway enhances mut p53 protein stability. SREBP2 degradation is regulated by GSK3, which is phosphorylated and inactivated by Akt, so p53 signaling will lead to Akt inhibition and increased SREBP2 degradation [101,102,103].

Overall, abnormal p53 promotes SREBP2 activity by upregulating the mevalonate pathway and enhancing SREBP2 stability, by inhibiting AMPK signaling and stimulating mTORC1 signaling (which drives SREBP2 activation) and by inhibiting Akt signaling (which reduces GSK3 activity and mature SREBP2 degradation. All mechanisms result in the accumulation of mature SREBP2, upregulation of SREBP2 targeted genes and upregulation of the mevalonate pathway [104].

### 7.2. AR (Androgen Receptor)

Androgens are essential for prostate gland development, but also play an important role in PC development and progression [105]. Androgen hormones, in order of potency, are: dihydrotestosterone (DHT), testosterone, androstenedione and dehydro-epianandrosterone. Serum testosterone originates from testis (95%) and adrenal gland (5%) and mostly is bound to sex hormone-binding globulin (SHBG) and albumin, while 1–2% is free. According to the free hormone hypothesis, only free form testosterone can enter prostate cells via free diffusion whereas bound androgens are inactive. Circulating DHT, whose concentration is 10 times less than testosterone, mainly originates from testosterone to DHT conversion in non-gonadal tissues or directly from testes and adrenals [106]. It has been shown that androgens bound to SHBG can be internalized via multiligand endocytic receptor megalin (encoded by LRP2, low density lipoprotein-related protein 2) whose protein expression is increased in PC when comparing to benign prostate cells, and certain polymorphisms appear to influence PC outcomes [107]. Around 95% of entered testosterone is converted to DHT by the enzyme 5α-reductase [91,106,108]. Testosterone and DHT bind toAR, a member of the nuclear hormone receptor superfamily, and activated AR translocates to the nucleus where it binds to the androgen responsive element (ARE) mediated with remodeling proteins and regulates expression of specific genes [71]. AR gene is regulated in response to androgen stimulation. Under high androgen conditions, AR recruits lysine specific demethylase LSD1 to AR binding site 2 leading to repression of its own expression and expression of androgen synthesis genes, DNA synthesis and cell cycle progression genes, while expression of lipid and protein anabolic genes is upregulated, therefore synthesis of prostatic fluid is maintained. Under low androgen condition, prostate cancer cells increase AR expression [93].

Mutations and amplifications of AR or its enhancer are rare in primary PC but frequent in CRPC [109]. Progression to CRPC is primarily driven by AR reactivation under low androgen conditions due to AR alternations, intra-tumoral androgen synthesis, increased coactivator expression, and activation of kinases which then activate AR or even ligand promiscuity [91,92,93]. CRPC can provide intra-tumoral androgen by testosterone derivatives conversion or de novo androgen synthesized from cholesterol [92]. AR is located in SCAP gene. By increasing SCAP expression and subsequent SCAP prevalence over its retention proteins, androgens direct SREBP2 to activation [68]. AR competes with LXR as coactivator [69]. Activated LXR induces expression of SULT2A1, sulfotransferase, that deactivates testosterone by sulfurization, which further cannot activate AR [70,71].

In summary, cholesterol can influence PC progression, serving as precursor of androgen and stimulating AR signaling leads to upregulated SCAP and SREBP2 activation. AR can also bypass this by stimulating mTOR pathway [87]. Beside this canonical signaling of AR, ligand-bound AR in cytoplasm can activate PI3K/AKT and Ras-Raf-MAPK/ERK cascade via rapid, non-genomic AR signaling.

### 7.3. PI3K/AKT/MTOR

mTOR is a serine/threonine protein kinase in mTOR Complex 1 (mTORC1) and 2 (mTORC2). mTOR, MLST8 and DEPTOR are core components, RPTOR and AKT1S1 are characteristic components of mTORC1, while RICTOR, MAPKAP1 and PRR5 are characteristic components of mTORC2 [110]. mTORC1 is a nutrient/energy/stress sensor which regulates cell growth and metabolism. It is a downstream target of PI3K/Akt. Activated by hormones and growth factors under nutrient-rich conditions, it promotes anabolic processes (lipid, nucleotide, and protein synthesis), inhibits glucose uptake, activates Foxk1 transcription factor, increases HIF1α and suppresses autophagy [111,112]. mTORC1’s mechanism of action is phosphorylation of S6K1, S6K2, 4E-BP1 and 4E-BP2 (stimulation of cap-dependent translation) as well as phosphorylation of other downstream targets [113]. mTORC2 is primarily regulated by PI3K signaling hormones and growth factors, and phosphorylates Akt, SGK and some PKC isoforms. mTORC2 effects actin skeleton, cell survival and growth [113,114].

In contrast to benign and peritumoral prostate tissue, in PC mTOR and S6K are more expressed, while mTOR signaling is hyperactivated. Besides phosphorylation in cytoplasm, mTOR binds to DNA in the nucleus and directly interacts with the chromatin [115]. mTOR supports SREBP2 activation through two mechanisms: inhibiting autophagy and preventing the entry of Lipin-1 in the nucleus. Inactive mTORC1 allows autophagosome and endosome delivery to lysosomes that become loaded with membrane cholesterol. Consequently, cholesterol in ER also increases and suppresses SREBP2. Active mTORC1 inhibits autophagy while stimulating endosome recycling, thus the content of cholesterol in ER is reduced and consequently SREBP2 is activated [116]. Activated mTOR phosphorylates Lipin-1 which then translocates to cytoplasm and functions as phosphatide phosphatase. mTOR inhibition results in Lipin-1 translocation to the nucleus which, by promoting SREBP binding to the nuclear matrix, impairs SREBP binding to target genes [112,117]. PI3K/Akt/mTOR signaling is antagonized by the tumor suppressor phosphatases and tensin homolog (PTEN) [118]. PTEN deletions and/or mutations are present in up to 30% of primary and 63% of metastatic PC [119,120]. PTEN loss upregulates PI3K/AKT/mTOR pathway which in turn upregulates SREBP and LDLR in PC cells, which leads to androgen independent accumulation of cholesteryl ester in high-grade and metastatic human PC [87]. PTEN, via AKT and GSK3β phosphorylation, labels SREBP2 and SMARCA4 for degradation. PTEN loss stabilizes SREBP2 and SMARCA4. SMARCA4 is part of the chromatin remodeling complex effecting a wide range of genes. Increased SMARCA4 in PTEN-deficient PC cells leads to chromatin remodeling and a pro-tumorigenic transcriptome, including increased c-Myc and phospho-ERK [121]. Furthermore, AR pathway can be detoured by the mTOR pathway [87]. Ligand-bound AR activates cytoplasmic mTOR function and increases mTOR translocation to the nucleus. Nuclear mTOR protein levels positively correlate with PC progression. It has been shown that 35% of androgen regulated genes are mTOR-mediated including mTOR-chromatin binding and cholesterol biosynthesis genes ACLY, ADH1A, ADRM1 and AGTRAP. AR transcriptional change in PC metabolism is accomplished via mTOR chromatin-binding, but in CRPC mTOR metabolic reprogramming is also present in the absence of androgen [115].

In summary, mTOR decreases cholesterol level in ER, which induces SREBP2 translocation, and allows SREBP2-DNA association. PTEN loss leads to constitutive mTOR activation, chromatin remodeling and transcriptions of oncogenes. A study on mouse prostates revealed that loss of PTEN alone leads to indolent tumors, but is not sufficient for PC progression. High-fat diet (HFD) stimulated lipid accumulation or additional gene alternation must be present [122].

### 7.4. MAPK

MAPK signaling pathway is frequently deregulated in advanced PC and its negative regulator PML is commonly co-deleted with PTEN. In PTEN-null mouse prostates, PML loss reactivates MAPK and promotes indolent PC into metastatic PC while SREBP2 regulated genes in PTEN and PML double-null mouse PC are upregulated on mRNA and protein level [122]. SREBP2 is a direct target of the ERK, a subfamily of MAPK. Since MAPKs phosphorylates SREBP2 near sumoylation site, it competes with sumoylation which result in reversed SUMO-induced attenuation of SREBP2 transcriptional activity [59]. To conclude, MAPK pathway via ERK increases SREBP2 activity. 

## 8. Acidity

PC progression is followed by complex tumor microenvironment transformation that plays a critical role in PC pathogenesis and promotion. Low oxygen condition, excessive glycolysis, carbonic anhydrase overexpression and reduced blood supply contribute to extracellular acidosis [123] which, in PC culture further induces increased extracellular vesicles (size: 110–180 nm) production which express higher PSA level. Amount of EV in plasma of PC patients is two to three-fold higher than in BHP or control patients and is homogenous and smaller in size (125–180 nm) [124]. Acid extracellular pH attenuated the cytotoxicity of daunorubicin on PC cells [125]. A study on the pancreatic cell line revealed that extracellular acidic pH (pH 6.8) lowered intracellular pH and triggered SREBP2 activation and upregulated cholesterol biosynthetic genes and acetyl-CoA synthetases-enzyme for acetate to acetyl-CoA conversion, which plays an important role in tumor cell growth under acidic pH [126].

## 9. SREBP2 Targets

Activated SREBP2 is a transcription factor that binds to SREs in promoters of target genes which activates gene expression of mevalonate pathway enzymes and enhances LDL uptake [48]. Increased cholesterol levels allow membrane biogenesis, de novo androgen synthesis and activation of AR signaling independently of androgens in the blood [127].

**Figure 2:**
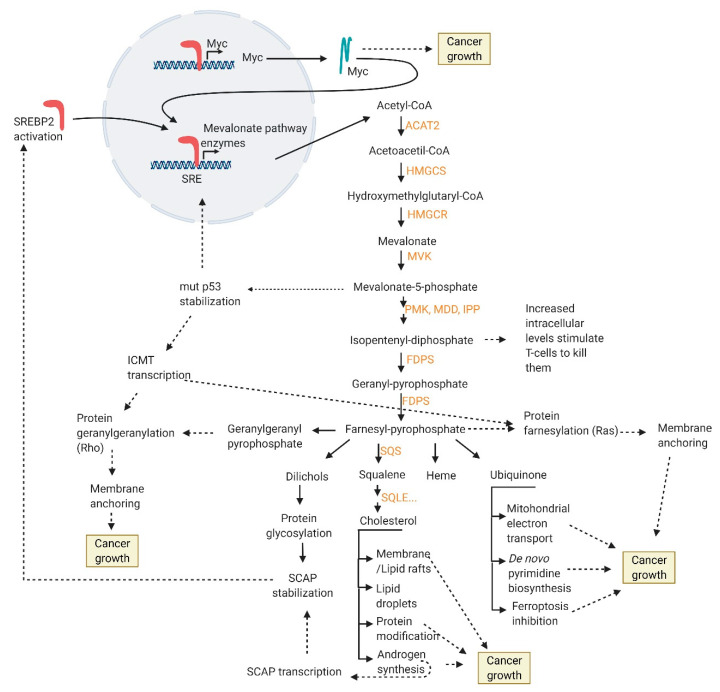
Cholesterol biosynthesis intermediates and products support prostate cancer growth. Enzymes involved in de novo cholesterol synthesis are indicated in orange. Acetyl-CoA acetyltransferase 2 (ACAT2), 3-hydroxy-3-methylglutaryl-CoA synthase (HMGCS), 3-hydroxy-3-methylglutaryl-CoA reductase (HMGCR), mevalonate kinase (MVK), phosphomevalonate kinase (PMK), diphospho-mevalonate decarboxylase(MDD), Isopentenyl-pyrophosphate isomerase (IPP), farnesyl-diphosphate synthase (FDPS), squalene synthase (SQS), squalene monooxygenase(SQLE), sterol regulatory element–binding protein-2 (SREBP2), sterol response element (SRE), isoprenyl-cysteine carboxyl methyltransferase (ICMT). Created with BioRender.com (accessed on 5 September 2021).

Mevalonate metabolism provides cholesterol, mevalonate-5-phosphate, ubiquinone, dolichols, heme, isopentenyl-diphosphate, FPP and GGPP, which supports PC growth and metastases (Figure 2).

Mevalonate-5-phosphate stabilizes mut p53 which further inactivates wild-type p53 and, by binding to SREBP2, upregulates mevalonate pathway gene expression. Ubiquinone (also known as coenzyme Q10) supports mitochondrial electron transport and de novo pyrimidine biosynthesis. Oxidoreductase FSP1, a strong suppressor of ferroptosis, reduces ubiquinone to generate lipophilic radical-trapping antioxidant and thus blocks ferroptosis, iron-dependent nonapoptotic cell death caused by lipids peroxidation [128,129]. Dolichols participate in protein glycosylation. Glucose- derived N-glycosylation of SCAP protects it from proteolysis and this modification is necessary for SCAP/SREBP exit from the ER [130]. Heme is in the prostatic group of hemoproteins. Free, unbound heme (newly synthesized or dissociated form hemoproteins) stimulate ROS production [131]. Isopentenyl-diphosphate is generated only by the mevalonate pathway. Increased intracellular levels stimulate T-cells to kill them [132]. Farnesyl and geranyl groups are used for G protein prenylation, which enables them to anchor to the membrane and signal transduction. Ras and Rho are known oncogenes that are prenylated [133]. Increased cholesterol and fatty acids lead to increase lipid rafts formation and subsequently activation of PI3K/AKT/mTOR pathway in lipid rafts [134]. Targeting SREBP2 activity alters composition of cell membrane and inhibits signal transduction in lipid rafts [135]. SREBP2 promotes tumorigenicity and stemness of PC cells through direct interaction with promoter of Myc-main downstream target of SREBP2 in cancer regulation, while mTORC1 enhances Myc translation efficiency [136]. MYC interacts with mevalonate pathway gene promotors near SRE [137,138].

To conclude, SREBP2 supports tumor growth by providing cholesterol as building block for membrane synthesis, lipid rafts and androgen synthesis, while the via mevalonate pathway provides molecules required for molecular membrane docking and modification, ferroptosis inhibition, energy, and nucleotide production. 

## 10. Cholesterol-Lowering Drugs and HDL Particles 

Statins are clinically approved cholesterol-lowering drugs for primary and secondary cardiovascular disease prevention. Many studies have confirmed the beneficial effect of statins on PC, but there are also disagreements [138]. Numerous studies have linked statins with reduced risk of PC, especially advanced and lethal PC, reduced risk of BCR after radical prostatectomy and radiotherapy, and prolonged time to progression in patients on androgen deprivation therapy [139,140,141]. A comprehensive study performed on 68,432 men with PC showed that use of statins before a PC diagnosis was associated with reduced risk of PC-specific mortality. After considering surrogate indicators of preventive care (PSA and cholesterol screening rate), the effect of statin use was abolished. This points out that careful interpretation of results is required as they can be confounded [142]. Nevertheless, results of in vitro and in vivo experiments provide a deeper molecular insight into conflicting epidemiologic studies.

Statins are competitive inhibitors of HMGCR, the rate-limiting enzyme in mevalonate pathway. Mevalonate-5-phosphate, an intermediate in the mevalonate pathway, stabilizes conformational or misfolded mut p53 which further upregulates expression of mevalonate pathway enzymes. By inhibiting the mevalonate pathway, statins reduce mut p53 generation, FPP and GGPP production, which leads to reduced prenylation of Ras and Rho [99,143,144]. Besides blocking the binding pocket in HMGCR, some statins target other molecules important for prostate cancer and show potential as an anti-cancer agent (listed in Table 1). Statins induce mut p53 degradation by disrupting its interaction with DNAJA1. DNAJA1 chaperone overexpression and ubiquitin ligase CHIP downregulation diminishes effect of statins. Impact on wild type p53 or DNA contact mutp53 with native conformation is minimal [143]. Few statins are also substrates of organic anionic transporter SLCO2B1, hence competitively reduce the uptake of androgen dehydroepiandrosterone sulfate, which is a precursor to more potent androgens [139]. 

Fatostatin is a drug that by binding to SCAP inhibits SCAP/SREBP dissociation from INSIG. Since this step blocks activation of SREBP1 and SREBP2, and the effect of fatostatin reflects cholesterogenesis and lipogenesis. It also inhibited tubulin polymerization, inhibited cell proliferation and induced apoptotic death in androgen-responsive and androgen-insensitive PC cells, as well as in metastatic PC cell lines, especially the one with mutant p53 [98,135,145]. Two months long treatment with fatostatin inhibited PC proliferation and distant lymph node metastasis in a genetically engineered mouse model of PC driven by Pten and Pml co-deletion [122]. Betulin and xanthohumol are natural SCAP/SREBP translocation inhibitors. Betulin induces SCAP-INSIGN interaction while Xanthohumol blocks SREBP incorporation in COPII vesicle [146,147]. Cell culture experiments showed that their PC suppression influence is associated with interaction with other molecules rather than SREBP [148,149,150].

Inhibition of HMGCR and consequently intracellular sterol pool depletion by statins leads to feedback activation of SREBP cleavage so therapies simultaneously inhibit mevalonate pathway and SREBP2 activation are in progress. Dipyridamole, an FDA approved drug for cerebral ischemia, inhibits the feedback loop, cooperates with statins and induces apoptosis in hematologic malignancies. Furthermore, dipyridamole potentiates the effect of statins, so less statin concentration is required [84,151,152].

Docetaxel provides first-class chemotherapy for the currently considered incurable metastatic CRPC. This tubulin stabilizing drug reduces AR nuclear translocation. Itwas shown that DHT reduces tubulin protein expression in PC [153]. The benefit of this chemotherapy is short-term and resistance connected with p53 mutation soon develops. Fatostatin synergizes with docetaxel for greater proliferation inhibition and apoptosis induction in PC, in vitro and in vivo mouse xenografts, particularly in high mut p53 PC. This combination might improve existing therapy for aggressive PC [98]. *HMGCR* expression affects enzalutamide sensitivity. In enzalutamide-resistant cell lines, HMGCR is overexpressed, and its activity increases after enzalutamide treatment. Simvastin restored sensitivity to enzalutamide. Combination of simvastin and enzalutamide significantly inhibited proliferation and induced, potentially mTOR mediated, maximum degradation of AR [74].Statins differ in their lipophilicity, dose, and effect on lipid profile [154,155]. Concentrations of statins used in vitro are much higher (mM range) than concentration in plasma of statin users (nM range). Although high dosage statins in PC cell line exhibited an anti-proliferative effect, low dosage of statins showed no effect on proliferation and migration but elevated self-renewal capacity and anchorage-independent growth [156]. Longer high dose of atorvastatin (80 mg) taken in the period between prostate biopsy and radical prostatectomy decreased proliferation of high-grade PC while fluvastatin (80 mg) increased apoptosis [157,158]. Besides repurposing cholesterol-lowering drugs into anticancer agents, HDL particles have qualities for improving drug delivery. Natural HDL particles are small (11 nm), deliver ssRNA and act via SR-B1 receptor, and thus avoid lysosomal processing. SR-B1 is overexpressed in PC, while minimally expressed in normal prostate cells. These characteristics make HDL particles suitable for drug delivery (chemotherapeutics, siRNA) via SR-B1 receptor. Encapsulating paclitaxel in reconstituted HDL postponed its degradation and increased selective drug delivery, which resulted in increased in vitro cytotoxicity and in vivo tolerance in comparison to free paclitaxel [159,160]. 

Mimics of spherical HDL loaded with siRNA targeting AR reduced AR mRNA in androgen-sensitive and androgen-insensitive PC cell lines. Systemic AR knockdown in mice, achieved with particles targeting *AR* mRNA, reduced cell viability in vitro and in vivo (xenograft) following reduction of hematocrit, white blood cells and neutrophils [161]. Valrubicin, incorporated in reconstituted high-density lipoprotein enhanced with superparamagnetic iron oxide nanoparticle, showed increased cytotoxicity of PC cell line and increased survival of normal prostate cell line in comparison to free valrubicin [162]. Although these results seem promising, off-target accumulation occurs in liver and spleen due to -B1 expression, thus SR-B1 targeting should be combined with additional targeting (e.g., another receptor, pH-sensitive nanoparticle, photosensitive porphyrin nanoparticle) to increase selectiveness [159]. 

## 11. Perspective

Scientists have made remarkable effort to understand the mechanisms behind PC etiopathogenesis. While the achievements in vitro and xenograft experiments may seem encouraging, PC is still the second most common cancer worldwide [163]. A vast amount of research has been done on PC cell lines, particularly on LNCaP. This cell line was derived from the supraclavicular lymph node biopsy 40 years ago from a PC patient after oral estrogen uptake, orchiectomy and estramustine exposure (strong mutagen). It is null for the PTEN gene and has hypo-tetraploid karyotype. Cell lines are clonal while PC is heterogeneous in cancer clones and cell types. Cell lines are dependent on supplied growth factors but signaling networks not used by the cell often degrades or mutates. Therefore, researchers need a more representative PC model [164]. Gene expression analysis in healthy and cancer tissue samples is also common. Expression data examination of seven patient cohorts showed that tissue heterogeneity is usually neglected, and stroma is more commonly represented in healthy tissue samples. Consequently, cholesterol synthesis genes are confounded by stroma tissue. When comparing healthy epithelial cells and cancer cells with balanced stroma content, cholestero-genic genes are downregulated in primary PC. Still, the limitation of this study is in not distinguishing reactive stroma from healthy stroma [44]. Furthermore, changes in transcriptome do not reflect directly on proteome. Gene copy number, DNA methylation, miRNA and mRNA expression do not consistently predict protein landscape, particularly in CRPC. This indicates that, due to dysfunctional regulatory systems in CRPC, many genetic and epigenetic changes arise that do not impact protein level [165].

According to studies done so far, it is evident that cholesterol has a significant role in PC. To understand and outwit PC, we need to base research on actual PC biopsies, primary cell culture and radical prostatectomy tissue. Furthermore, comprehensive analysis that would encompass genomic, transcriptomic, and proteomic data considering tissue homogeneity are required as well as stratification of patients according to stage, Gleason score, interindividual genetic diversity and adjuvant treatments, which would pave the way for a personalized approach and long-term treatments.

## 12. Conclusions

It is evident that cholesterol is an important PC growth supporter. Cholesterol homeostasis in the cell is balanced by SREBP2 and LXR activity, which is related to endoplasmic reticulum cholesterol level, INSIG/SCAP ratio, transcriptional modifications, post-translational modifications, and mevalonate pathway metabolites. Many deregulated pathways in PC are implicated in SREBP2 activation which can have an effect on:

- increased membrane/lipid rafts synthesis and protein prenylation which alter membrane composition and cell signaling
- mut p53 stabilization and further mevalonate pathway promotion
- nucleotide synthesis, mitochondrial electron transport, protein anchoring and stabilization, ferroptosis inhibition
- intracellular androgen synthesis
- SREBP2 interaction with Myc promoter

There are numerous data that arise from experiments on cell lines, animal models and human studies which take into consideration only a few variables. Still, comprehensive multi-omics analyses that would improve patient stratification and development of personalized medicine strategies are required. 

## Figures and Tables

**Table 1:** List of cholesterol-lowering drugs, their targets and cellular function with emphasize on particular statin type.

Drug	Target	Function	Reference
Statins (in general)	HMGCR	Reduces HMG-CoA to mevalonate	[143]
Atorvastatin	SLCO2B1	Androgen transporting gene (uptake)	[139]
	DNAJA1	Cochaperone protein (protects mut p53 form degradation)	[143]
Pravastatin	SLCO2B1	Androgen transporting gene (uptake)	[139]
Fatostatin	SCAP	SREBP2 and SREBP1 maturation	[145]
	Tubulin	Maintenance of microtubule organization	[145]
Lovastatin	DNAJA1	Cochaperone protein (protects mut p53 form degradation)	[143]
Mevastatin	DNAJA1	Cochaperone protein (protects mut p53 form degradation)	[143]
Betulin	SCAP-INSIGN interaction	SCAP/SREBP translocation	[147]
Xanthohumol	Sec23/24 (COPII vesicle)	SCAP/SREBP translocation	[146]
**Combination therapy**			
Statins + Dipyridamole (antiplatelet agent)	Phosphodiesterase	Hydrolysis of cyclic nucleotides	[84,152]
Simvastin + enzalutamide (antiandrogen)	AR	Activation of AR signaling pathway	[74]
Fatostatin + Docetaxel (chemotherapeutic agent)	Tubulin	Maintenance of microtubule organization	[98,153]

## Abbreviations

ABCA1	ATP-binding cassette transporter A1
ABCG1	ATP-binding cassette transporter G
ACLY	ATP citrate lyase
AMPK	AMP-activated protein kinase
AR	Androgen receptor
ARE	Androgen responsive element
BCR	Biochemical recurrence
BPH	Benign prostatic hyperplasia
COPII	Coatomer II
CRPC	Castrate-resistant prostate cancer
DHT	Dihydrotestosterone
EGFR	Epidermal growth factor receptor
ERK	Extracellular signal-regulated kinase
FA	Fatty acid
FDG-PET	Fluorodeoxyglucose-positron emission tomography
FPP	Farnesyl diphosphate
GGPP	Geranyl-geranyl-pyrophosphate
GSK3	Glycogen synthase kinase 3
HDL	High-density lipoprotein
HMG-CoA	3-hydroxy-3methylglutaryl-CoA
INSIG	Insulin-induced gene
ICMT	Isoprenylcysteine carboxyl methyltransferase
LDL	Low-density lipoprotein
LDLR	Low-density lipoprotein receptor
LRP8	Lipoprotein receptor-related proteins 8
LXR	Liver X receptor
MAPK	Mitogen-activated protein kinase
mTOR	Mechanistic target of rapamycin
PC	Prostate cancer
PSA	Prostate-specific antigen
ROS	Reactive oxygen species
S1P	Site-1 protease
S2P	Site-2 protease
SCAP	SREBP cleavage-activating protein
SHBG	Sex hormone-binding globulin
SQLE	Squalene monooxygenase
SR-B1	Scavenger receptor class B member 1
SRE	Sterol response element
SREBP2	Sterol regulatory element–binding protein-2

## References

[1] Krušlin B., Škara L., Vodopić T., Vrhovec B., Murgić J., Štimac G., Fröbe A., Lež C., Ulamec M., Gall-Trošelj K. (2021). Genetics of Prostate Carcinoma. Acta Med. Acad..

[2] Abramovic I., Vrhovec B., Skara L., Vrtaric A., Gabaj N.N., Kulis T., Stimac G., Ljiljak D., Ruzic B., Kastelan Z. (2021). MiR-182-5p and miR-375-3p Have Higher Performance Than PSA in Discriminating Prostate Cancer from Benign Prostate Hyperplasia. Cancers.

[3] Culp M.B., Soerjomataram I., Efstathiou J.A., Bray F., Jemal A. (2020). Recent Global Patterns in Prostate Cancer Incidence and Mortality Rates. Eur. Urol..

[4] Adamaki M., Zoumpourlis V. (2021). Immunotherapy as a Precision Medicine Tool for the Treatment of Prostate Cancer. Cancers.

[5] Milliron B., Bruneau M., Obeid E., Gross L., Bealin L., Smaltz C., Giri V.N. (2019). Diet assessment among men undergoing genetic counseling and genetic testing for inherited prostate cancer: Exploring a teachable moment to support diet intervention. Prostate.

[6] Hager M.H., Solomon K.R., Freeman M.R. (2006). The role of cholesterol in prostate cancer. Curr. Opin. Clin. Nutr. Metab. Care..

[7] Moon H., Ruelcke J., Choi E., Sharpe L., Nassar Z., Bielefeldt-Ohmann H., Parat M.-O., Shah A., Francois M., Inder K.L. (2015). Diet-Induced Hypercholesterolemia Promotes Androgen-Independent Prostate Cancer Metastasis via IQGAP1 and Caveolin-1. Oncotarget.

[8] Whittemore A.S., Kolonel L.N., Wu A.H., John E.M., Gallagher R.P., Howe G.R., Burch J.D., Hankin J., Dreon D.M., West D.W. (1995). Prostate Cancer in Relation to Diet, Physical Activity, and Body Size in Blacks, Whites, and Asians in the United States and Canada. J. Natl. Cancer Inst..

[9] Buszewska-Forajta M., Pomastowski P., Monedeiro F., Walczak-Skierska J., Markuszewski M., Matuszewski M., Markuszewski M.J., Buszewski B. (2021). Lipidomics as a Diagnostic Tool for Prostate Cancer. Cancers.

[10] Vaidyanathan V., Karunasinghe N., Jabed A., Pallati R., Kao C.H.-J., Wang A., Marlow G., Ferguson L.R. (2016). Prostate Cancer: Is It a Battle Lost to Age?. Geriatrics.

[11] Pelton K., Freeman M.R., Solomon K.R. (2012). Cholesterol and prostate cancer. Curr. Opin. Pharmacol..

[12] Singh G., Sankanagoudar S., Dogra P., Chandra N.C. (2017). Interlink between cholesterol & cell cycle in prostate carcinoma. Indian J. Med. Res..

[13] Solomon K.R., Pelton K., Boucher K., Joo J., Tully C., Zurakowski D., Schaffner C.P., Kim J., Freeman M.R. (2009). Ezetimibe Is an Inhibitor of Tumor Angiogenesis. Am. J. Pathol..

[14] Uo T., Sprenger C.C., Plymate S.R. (2020). Androgen Receptor Signaling and Metabolic and Cellular Plasticity during Progression to Castration Resistant Prostate Cancer. Front. Oncol..

[15] Mah C.Y., Nassar Z.D., Swinnen J.V., Butler L.M. (2020). Lipogenic Effects of Androgen Signaling in Normal and Malignant Prostate. Asian J. Urol..

[16] Costello L.C., Franklin R.B. (2016). A Comprehensive Review of the Role of Zinc in Normal Prostate Function and Metabolism; and Its Implications in Prostate Cancer. Arch. Biochem. Biophys..

[17] Costello L.C., Feng P., Milon B., Tan M., Franklin R.B. (2004). Role of zinc in the pathogenesis and treatment of prostate cancer: Critical issues to resolve. Prostate Cancer Prostatic Dis..

[18] Bader D.A., McGuire S.E. (2020). Tumour metabolism and its unique properties in prostate adenocarcinoma. Nat. Rev. Urol..

[19] Zadra G., Ribeiro C.F., Chetta P., Ho Y., Cacciatore S., Gao X., Syamala S., Bango C., Photopoulos C., Huang Y. (2018). Inhibition of de novo Lipogenesis Targets Androgen Receptor Signaling in Castration-Resistant Prostate Cancer. Proc. Natl. Acad. Sci. USA.

[20] Bertilsson H., Tessem M.-B., Flatberg A., Viset T., Gribbestad I., Angelsen A., Halgunset J. (2012). Changes in Gene Transcription Underlying the Aberrant Citrate and Choline Metabolism in Human Prostate Cancer Samples. Clin. Cancer Res..

[21] Gonzalez-Menendez P., Hevia D., Mayo J.C., Sainz R.M. (2018). The dark side of glucose transporters in prostate cancer: Are they a new feature to characterize carcinomas?. Int. J. Cancer.

[22] Krycer J.R., Brown A.J. (2013). Cholesterol Accumulation in Prostate Cancer: A Classic Observation from a Modern Perspective. Biochim. Biophys. Acta-Rev. Cancer.

[23] Poulose N., Mills I.G., Steele R.E. (2018). The impact of transcription on metabolism in prostate and breast cancers. Endocr. Relat. Cancer..

[24] Yupeng L., Yuxue Z., Pengfei L., Cheng C., Yashuang Z., Dapeng L., Chen D. (2015). Cholesterol Levels in Blood and the Risk of Prostate Cancer: A Meta-analysis of 14 Prospective Studies. Cancer Epidemiol. Biomark. Prev..

[25] Huang B., Song B., Xu C. (2020). Cholesterol metabolism in cancer: Mechanisms and therapeutic opportunities. Nat. Metab..

[26] Likus W., Siemianowicz K., Bieńk K., Pakuła M., Pathak H., Dutta C., Wang Q., Shojaei S., Assaraf Y.G., Ghavami S. (2016). Could drugs inhibiting the mevalonate pathway also target cancer stem cells?. Drug Resist. Updat. Rev. Comment Antimicrob. Anticancer Chemother..

[27] Hryniewicz-Jankowska A., Augoff K., Sikorski A.F. (2019). Highlight article: The role of cholesterol and cholesterol-driven membrane raft domains in prostate cancer. Exp. Biol. Med. (Maywood).

[28] Li H., Feng Z., He M.-L. (2020). Lipid Metabolism Alteration Contributes to and Maintains the Properties of Cancer Stem Cells. Theranostics.

[29] Matsushita Y., Nakagawa H., Koike K. (2021). Lipid Metabolism in Oncology: Why It Matters, How to Research, and How to Treat. Cancers.

[30] Brown A.J. (2007). Cholesterol, Statins and Cancer. Clin. Exp. Pharmacol. Physiol..

[31] Ding X., Zhang W., Li S., Yang H. (2019). The role of cholesterol metabolism in cancer. Am. J. Cancer Res..

[32] Luo J., Jiang L.-Y., Yang H., Song B.-L. (2019). Intracellular Cholesterol Transport by Sterol Transfer Proteins at Membrane Contact Sites. Trends Biochem. Sci..

[33] Blanco A., Blanco G., Blanco A., Blanco G. (2017). Chapter 15-Lipid Metabolism.

[34] Shapiro M.D., Tavori H., Fazio S. (2018). PCSK9: From Basic Science Discoveries to Clinical Trials. Circ. Res..

[35] Luo J., Yang H., Song B.-L. (2020). Mechanisms and regulation of cholesterol homeostasis. Nat. Rev. Mol. Cell Biol..

[36] Shen W.-J., Azhar S., Kraemer F.B. (2018). SR-B1: A Unique Multifunctional Receptor for Cholesterol Influx and Efflux. Annu. Rev. Physiol..

[37] Traughber C.A., Opoku E., Brubaker G., Major J., Lu H., Lorkowski S.W., Neumann C., Hardaway A., Chung Y.-M., Gulshan K. (2020). Uptake of high-density lipoprotein by scavenger receptor class B type 1 is associated with prostate cancer proliferation and tumor progression in mice. J. Biol. Chem..

[38] Cha J.-Y., Lee H.-J. (2016). Targeting Lipid Metabolic Reprogramming as Anticancer Therapeutics. J. Cancer Prev..

[39] Schug Z.T., Peck B., Jones D.T., Zhang Q., Grosskurth S., Alam I.S., Goodwin L.M., Smethurst E., Mason S., Blyth K. (2015). Acetyl-CoA Synthetase 2 Promotes Acetate Utilization and Maintains Cancer Cell Growth Under Metabolic Stress. Cancer Cell.

[40] Giacomini I., Gianfanti F., Desbats M.A., Orso G., Berretta M., Prayer-Galetti T., Ragazzi E., Cocetta V. (2021). Cholesterol Metabolic Reprogramming in Cancer and Its Pharmacological Modulation as Therapeutic Strategy. Front. Oncol..

[41] Afonso M.S., Machado R.M., Lavrador M.S., Quintao E.C.R., Moore K.J., Lottenberg A.M. (2018). Molecular Pathways Underlying Cholesterol Homeostasis. Nutrients.

[42] Gesto D.S., Pereira C.M.S., Cerqueira N.M.F.S., Sousa S.F. (2020). An Atomic-Level Perspective of HMG-CoA-Reductase: The Target Enzyme to Treat Hypercholesterolemia. Molecules.

[43] Alioui A., Celhay O., Baron S., Lobaccaro J.-M.A. (2014). Lipids and Prostate Cancer Adenocarcinoma. Clin. Lipidol..

[44] Rye M.B., Bertilsson H., Andersen M.K., Rise K., Bathen T.F., Drabløs F., Tessem M.-B. (2018). Cholesterol synthesis pathway genes in prostate cancer are transcriptionally downregulated when tissue confounding is minimized. BMC Cancer.

[45] Wang D.Q.-H. (2007). Regulation of intestinal cholesterol absorption. Annu. Rev. Physiol..

[46] Repa J.J., Mangelsdorf D.J. (2000). The role of orphan nuclear receptors in the regulation of cholesterol homeostasis. Annu. Rev. Cell Dev. Biol..

[47] Cheng C., Ru P., Geng F., Liu J., Yoo J.Y., Wu X., Cheng X., Euthine V., Hu P., Guo J.Y. (2015). Glucose-Mediated N-glycosylation of SCAP Is Essential for SREBP-1 Activation and Tumor Growth. Cancer Cell.

[48] Xue L., Qi H., Zhang H., Ding L., Huang Q., Zhao D., Wu B.J., Li X. (2020). Targeting SREBP-2-Regulated Mevalonate Metabolism for Cancer Therapy. Front. Oncol..

[49] Rawson R.B., DeBose-Boyd R., Goldstein J.L., Brown M.S. (1999). Failure to cleave sterol regulatory element-binding proteins (SREBPs) causes cholesterol auxotrophy in Chinese hamster ovary cells with genetic absence of SREBP cleavage-activating protein. J. Biol. Chem..

[50] Gao Y., Zhou Y., Goldstein J.L., Brown M.S., Radhakrishnan A. (2017). Cholesterol-induced conformational changes in the sterol-sensing domain of the Scap protein suggest feedback mechanism to control cholesterol synthesis. J. Biol. Chem..

[51] Radhakrishnan A., Goldstein J.L., McDonald J.G., Brown M.S. (2008). Switch-like Control of SREBP-2 Transport Triggered by Small Changes in ER Cholesterol: A Delicate Balance. Cell Metab..

[52] Lee J.N., Song B., DeBose-Boyd R.A., Ye J. (2006). Sterol-regulated degradation of Insig-1 mediated by the membrane-bound ubiquitin ligase gp78. J. Biol. Chem..

[53] Xiao-Ying D., Sheng-Qiu T., Jin-Ding C. (2012). Dual functions of Insig proteins in cholesterol homeostasis. Lipids. Health Dis..

[54] Kuan Y.-C., Takahashi Y., Maruyama T., Shimizu M., Yamauchi Y., Sato R. (2020). Ring Finger Protein 5 Activates Sterol Regulatory Element–Binding Protein 2 (SREBP2) to Promote Cholesterol Biosynthesis via Inducing Polyubiquitination of SREBP Chaperone SCAP. J. Biol. Chem..

[55] Fan Z., Kong M., Li M., Hong W., Fan X., Xu Y. (2020). Brahma Related Gene 1 (Brg1) Regulates Cellular Cholesterol Synthesis by Acting as a Co-factor for SREBP2. Front. cell Dev. Biol..

[56] Matsuzaka T., Shimano H. (2013). Insulin-dependent and -independent regulation of sterol regulatory element-binding protein-1c. J. Diabetes Investig..

[57] Sundqvist A., Bengoechea-Alonso M.T., Ye X., Lukiyanchuk V., Jin J., Harper J.W., Harper J., Ericsson J. (2005). Control of Lipid Metabolism by Phosphorylation-Dependent Degradation of the SREBP Family of Transcription Factors by SCFFbw7. Cell Metab..

[58] Giandomenico V., Simonsson M., Grönroos E., Ericsson J. (2003). Coactivator-Dependent Acetylation Stabilizes Members of the SREBP Family of Transcription Factors. Mol. Cell Biol..

[59] Arito M., Horiba T., Hachimura S., Inoue J., Sato R. (2008). Growth factor-induced phosphorylation of sterol regulatory element-binding proteins inhibits sumoylation, thereby stimulating the expression of their target genes, low density lipoprotein uptake, and lipid synthesis. J. Biol. Chem..

[60] Elhanati S., Kanfi Y., Varvak A., Roichman A., Carmel-Gross I., Barth S., Gibor G., Cohen H.Y. (2013). Multiple Regulatory Layers of SREBP1/2 by SIRT6. Cell Rep..

[61] Dufour J., Viennois E., De Boussac H., Baron S., Lobaccaro J.-M. (2012). Oxysterol receptors, AKT and prostate cancer. Curr. Opin. Pharmacol..

[62] Radhakrishnan A., Ikeda Y., Kwon H.J., Brown M.S., Goldstein J.L. (2007). Sterol-Regulated Transport of SREBPs from Endoplasmic Reticulum to Golgi: Oxysterols Block Transport by Binding to INSIG. Proc. Natl. Acad. Sci. USA.

[63] Scotti E., Calamai M., Goulbourne C.N., Zhang L., Hong C., Lin R.R., Choi J., Pilch P.F., Fong L.G., Zou P. (2013). IDOL Stimulates Clathrin-Independent Endocytosis and Multivesicular Body-Mediated Lysosomal Degradation of the Low-Density Lipoprotein Receptor. Mol. Cell. Biol..

[64] Calkin A.C., Tontonoz P. (2012). Transcriptional integration of metabolism by the nuclear sterol-activated receptors LXR and FXR. Nat. Rev. Mol. Cell Biol..

[65] Wang Y., Rogers P.M., Su C., Varga G., Stayrook K.R., Burris T.P. (2008). Regulation of Cholesterologenesis by the Oxysterol Receptor, LXRalpha. J. Biol. Chem..

[66] Mok E.H.K., Lee T.K.W. (2020). The Pivotal Role of the Dysregulation of Cholesterol Homeostasis in Cancer: Implications for Therapeutic Targets. Cancers.

[67] Yoshioka H., Coates H.W., Chua N.K., Hashimoto Y., Brown A.J., Ohgane K. (2020). A Key Mammalian Cholesterol Synthesis Enzyme, Squalene Monooxygenase, is Allosterically Stabilized by Its Substrate. Proc. Natl. Acad. Sci. USA.

[68] Heemers H., Verrijdt G., Organe S., Claessens F., Heyns W., Verhoeven G., Swinnen J. (2004). Identification of an Androgen Response Element in Intron 8 of the Sterol Regulatory Element-binding Protein Cleavage-activating Protein Gene Allowing Direct Regulation by the Androgen Receptor. J. Biol. Chem..

[69] Krycer J.R., Brown A.J. (2011). Cross-talk between the androgen receptor and the liver X receptor: Implications for cholesterol homeostasis. J. Biol. Chem..

[70] Runge-Morris M., Kocarek T.A., Falany C.N. (2013). Regulation of the Cytosolic Sulfotransferases by Nuclear Receptors. Drug Metab Rev..

[71] Lee J.H., Gong H., Khadem S., Lu Y., Gao X., Li S., Zhang J., Xie W. (2008). Androgen Deprivation by Activating the Liver X Receptor. Endocrinology.

[72] Lee H.J., Li J., Vickman R.E., Li J., Liu R., Durkes A.C., Elzey B.D., Yue S., Liu X., Ratliff T.L. (2018). Cholesterol Esterification Inhibition Suppresses Prostate Cancer Metastasis by Impairing the Wnt/β-catenin Pathway. Mol. Cancer Res..

[73] Asare G.A., Owusu-Boateng E., Asiedu B., Amoah B.Y., Essendoh E., Otoo R.Y. (2019). Oxidised low-density lipoprotein, a possible distinguishing lipid profile biomolecule between prostate cancer and benign prostatic hyperplasia. Andrologia..

[74] Kong Y., Cheng L., Mao F., Zhang Z., Zhang Y., Farah E., Bosler J., Bai Y., Ahmad N., Kuang S. (2018). Inhibition of cholesterol biosynthesis overcomes enzalutamide resistance in castration-resistant prostate cancer (CRPC). J. Biol. Chem..

[75] Jamnagerwalla J., Howard L.E., Allott E.H., Vidal A.C., Moreira D.M., Castro-Santamaria R., Andriole G.L., Freeman M.R., Freedland S.J. (2018). Serum cholesterol and risk of high-grade prostate cancer: Results from the REDUCE study. Prostate Cancer Prostatic Dis..

[76] Zapata D., Howard L.E., Allott E.H., Hamilton R.J., Goldberg K., Freedland S.J. (2015). Is PSA related to serum cholesterol and does the relationship differ between black and white men?. Prostate.

[77] Singh G., Sankanagoudar S., Dogra P.N., Mathur S.R., Chandra N.C. (2014). A study on lipid profile in prostate carcinoma patients admitted in AIIMS, New Delhi. J. Biomed Pharm. Res..

[78] Henriksson P., Ericsson S., Stege R., Eriksson M., Rudling M., Berglund L., Angelin B. (1989). Hypocholesterolaemia and Increased Elimination of Low-Density Lipoproteins in Metastatic Cancer of the Prostate. Lancet.

[79] Tatidis L., Gruber A., Vitols S. (1997). Decreased feedback regulation of low density lipoprotein receptor activity by sterols in leukemic cells from patients with acute myelogenous leukemia. J. Lipid. Res..

[80] Gonçalves R.P., Rodrigues D.G., Maranhão R.C. (2005). Uptake of High Density Lipoprotein (HDL) Cholesteryl Esters by Human Acute Leukemia Cells. Leuk. Res..

[81] Andreassen O.A., Zuber V., Thompson W.K., Schork A.J., Bettella F., Djurovic S., Desikan R.S., Mills I., Dale A.M., The PRACTICAL Consortium (2014). Shared common variants in prostate cancer and blood lipids. Int. J. Epidemiol..

[82] Chimento A., Casaburi I., Avena P., Trotta F., De Luca A., Rago V., Pezzi V., Sirianni R. (2019). Cholesterol and Its Metabolites in Tumor Growth: Therapeutic Potential of Statins in Cancer Treatment. Front. Endocrinol..

[83] Li X., Wu J.B., Li Q., Shigemura K., Chung L.W.K., Huang W.-C. (2016). SREBP-2 Promotes Stem Cell-Like Properties and Metastasis by Transcriptional Activation of c-Myc in Prostate Cancer. Oncotarget.

[84] Longo J., Mullen P.J., Yu R., van Leeuwen J.E., Masoomian M., Woon D.T., Wang Y., Chen E.X., Hamilton R.J., Sweet J.M. (2019). An Actionable Sterol-Regulated Feedback Loop Modulates Statin Sensitivity in Prostate Cancer. Mol. Metab..

[85] Lee B., Taylor M., Robinet P., Smith J.D., Schweitzer J., Sehayek E., Falzarano S.M., Magi-Galluzzi C., Klein E.A., Ting A.H. (2012). Dysregulation of Cholesterol Homeostasis in Human Prostate Cancer through Loss of ABCA1. Cancer Res..

[86] Schörghofer D., Kinslechner K., Preitschopf A., Schütz B., Röhrl C., Hengstschläger M., Stangl H., Mikula M. (2015). The HDL receptor SR-BI is associated with human prostate cancer progression and plays a possible role in establishing androgen independence. Reprod. Biol. Endocrinol..

[87] Yue S., Li J., Lee S.-Y., Lee H.J., Shao T., Song B., Cheng L., Masterson T.A., Liu X., Ratliff T. (2014). Cholesteryl ester accumulation induced by PTEN loss and PI3K/AKT activation underlies human prostate cancer aggressiveness. Cell Metab..

[88] Thysell E., Surowiec I., Hörnberg E., Crnalic S., Widmark A., Johansson A.I., Stattin P., Bergh A., Moritz T., Antti H. (2010). Metabolomic Characterization of Human Prostate Cancer Bone Metastases Reveals Increased Levels of Cholesterol. PLoS ONE.

[89] Chen Y., Hughes-Fulford M. (2001). Human prostate cancer cells lack feedback regulation of low-density lipoprotein receptor and its regulator, SREBP2. Int. J. Cancer.

[90] Ossoliński K., Nizioł J., Arendowski A., Ossolińska A., Ossoliński T., Kucharz J., Wiechno P., Ruman T. (2019). Mass spectrometry-based metabolomic profiling of prostate cancer-a pilot study. J. Cancer Metastasis Treat..

[91] Tan M.H.E., Li J., Xu H.E., Melcher K., Yong E. (2015). Androgen receptor: Structure, role in prostate cancer and drug discovery. Acta Pharmacol. Sin..

[92] Wilson S., Qi J., Filipp F.V. (2016). Refinement of the androgen response element based on ChIP-Seq in androgen-insensitive and androgen-responsive prostate cancer cell lines. Sci. Rep..

[93] Cai C., He H.H., Chen S., Coleman I., Wang H., Fang Z., Chen S., Nelson P.S., Liu X.S., Brown M. (2011). Androgen Receptor Gene Expression in Prostate Cancer Is Directly Suppressed by the Androgen Receptor Through Recruitment of Lysine-Specific Demethylase 1. Cancer Cell.

[94] Xiao Q., Sun Y., Dobi A., Srivastava S., Wang W., Srivastava S., Ji Y., Hou J., Zhao G.-P., Li Y. (2018). Systematic analysis reveals molecular characteristics of ERG-negative prostate cancer. Sci. Rep..

[95] Khemlina G., Ikeda S., Kurzrock R. (2015). Molecular Landscape of Prostate Cancer: Implications for Current Clinical Trials. Cancer Treat. Rev..

[96] Agell L., Hernández S., Salido M., De Muga S., Juanpere N., Arumi-Uria M., Menendez S., Lorenzo M., Lorente J.A., Serrano S. (2010). PI3K signaling pathway is activated by PIK3CA mRNA overexpression and copy gain in prostate tumors, but PIK3CA, BRAF, KRAS and AKT1 mutations are infrequent events. Mod. Pathol..

[97] Sun X., Huang J., Homma T., Kita D., Klocker H., Schafer G., Boyle P., Ohgaki H. (2009). Genetic alterations in the PI3K pathway in prostate cancer. Anticancer Res..

[98] Li X., Wu J.B., Chung L.W.K., Huang W.-C. (2015). Anti-cancer efficacy of SREBP inhibitor, alone or in combination with docetaxel, in prostate cancer harboring p53 mutations. Oncotarget.

[99] Parrales A., Thoenen E., Iwakuma T. (2018). The interplay between mutant p53 and the mevalonate pathway. Cell Death Differ..

[100] Borini Etichetti C.M., Arel Zalazar E., Cocordano N., Girardini J. (2020). Beyond the Mevalonate Pathway: Control of Post-Prenylation Processing by Mutant p53. Front. Oncol..

[101] Flöter J., Kaymak I., Schulze A. (2017). Regulation of Metabolic Activity by p53. Metabolites.

[102] Li Y., Xu S., Mihaylova M.M., Zheng B., Hou X., Jiang B., Park O., Luo Z., Lefai E., Shyy J.Y.-J. (2011). AMPK Phosphorylates and Inhibits SREBP Activity to Attenuate Hepatic Steatosis and Atherosclerosis in Diet-Induced Insulin-Resistant Mice. Cell Metab..

[103] Zhou G., Wang J., Zhao M., Xie T.-X., Tanaka N., Sano D., Patel A.A., Ward A.M., Sandulache V., Jasser S.A. (2014). Gain-of-Function Mutant p53 Promotes Cell Growth and Cancer Cell Metabolism via Inhibition of AMPK Activation. Mol. Cell.

[104] Kaymak I., Maier C.R., Schmitz W., Campbell A.D., Dankworth B., Ade C.P., Walz S., Paauwe M., Kalogirou C., Marouf H. (2019). Mevalonate Pathway Provides Ubiquinone to Maintain Pyrimidine Synthesis and Survival in p53-Deficient Cancer Cells Exposed to Metabolic Stress. Cancer Res..

[105] Kallio H.M.L., Hieta R., Latonen L., Brofeldt A., Annala M., Kivinummi K., Tammela T.L., Nykter M., Isaacs W.B., Lilja H.G. (2018). Constitutively active androgen receptor splice variants AR-V3, AR-V7 and AR-V9 are co-expressed in castration-resistant prostate cancer metastases. Br. J. Cancer.

[106] Marchetti P.M., Barth J.H. (2013). Clinical Biochemistry of Dihydrotestosterone. Ann. Clin. Biochem..

[107] Holt S.K., Karyadi D.M., Kwon E.M., Stanford J.L., Nelson P.S., Ostrander E.A. (2008). Association of Megalin Genetic Polymorphisms with Prostate Cancer Risk and Prognosis. Clin. Cancer Res..

[108] Li H., Pham T., McWhinney B.C., Ungerer J., Pretorius C., Richard D.J., Mortimer R.H., D’Emden M., Richard K. (2016). Sex Hormone Binding Globulin Modifies Testosterone Action and Metabolism in Prostate Cancer Cells. Int. J. Endocrinol..

[109] Huang Y., Jiang X., Liang X., Jiang G. (2018). Molecular and cellular mechanisms of castration resistant prostate cancer (Review). Oncol. Lett..

[110] Papadopoli D., Boulay K., Kazak L., Pollak M., Mallette F., Topisirovic I., Hulea L. (2019). mTOR as a central regulator of lifespan and aging. F1000Research.

[111] Valvezan A.J., Manning B.D. (2019). Molecular logic of mTORC1 signalling as a metabolic rheostat. Nat. Metab..

[112] Peterson T.R., Sengupta S.S., Harris T.E., Carmack A.E., Kang S.A., Balderas E., Guertin D.A., Madden K.L., Carpenter A., Finck B.N. (2011). mTOR Complex 1 Regulates Lipin 1 Localization to Control the SREBP Pathway. Cell.

[113] Ricoult S.J.H., Manning B.D. (2013). The multifaceted role of mTORC1 in the control of lipid metabolism. EMBO Rep..

[114] Yoon M.-S. (2017). The Role of Mammalian Target of Rapamycin (mTOR) in Insulin Signaling. Nutrients.

[115] Audet-Walsh É., Dufour C.R., Yee T., Zouanat F.Z., Yan M., Kalloghlian G., Vernier M., Caron M., Bourque G., Scarlata E. (2017). Nuclear mTOR acts as a transcriptional integrator of the androgen signaling pathway in prostate cancer. Genes Dev..

[116] Eid W., Dauner K., Courtney K.C., Gagnon A., Parks R.J., Sorisky A., Zha X. (2017). mTORC1 Activates SREBP-2 by Suppressing Cholesterol Trafficking to Lysosomes in Mammalian Cells. Proc. Natl. Acad. Sci. USA.

[117] Laplante M., Sabatini D.M. (2013). Regulation of mTORC1 and Its Impact on Gene Expression at a Glance. J. Cell Sci..

[118] Bitting R.L., Armstrong A.J. (2013). Targeting the PI3K/Akt/mTOR Pathway in Castration-Resistant Prostate Cancer. Endocr. Relat. Cancer.

[119] Lokody I.B., Francis J.C., Gardiner J.R., Erler J.T., Swain A. (2015). Pten Regulates Epithelial Cytodifferentiation during Prostate Development. PLoS ONE.

[120] Jillson L.K., Yette G.A., Laajala T.D., Tilley W.D., Costello J.C., Cramer S.D. (2021). Androgen Receptor Signaling in Prostate Cancer Genomic Subtypes. Cancers.

[121] Ding Y., Li N., Dong B., Guo W., Wei H., Chen Q., Yuan H., Han Y., Chang H., Kan S. (2019). Chromatin Remodeling ATPase BRG1 and PTEN are Synthetic Lethal in Prostate Cancer. J. Clin. Investig..

[122] Chen M., Zhang J., Sampieri K., Clohessy J., Mendez L., Gonzalez-Billalabeitia E., Liu X.-S., Lee Y.-R., Fung J., Katon J.M. (2018). An aberrant SREBP-dependent lipogenic program promotes metastatic prostate cancer. Nat. Genet..

[123] Huang S., Tang Y., Peng X., Cai X., Wa Q., Ren D., Li Q., Luo J., Li L., Zou X. (2016). Acidic extracellular pH promotes prostate cancer bone metastasis by enhancing PC-3 stem cell characteristics, cell invasiveness and VEGF-induced vasculogenesis of BM-EPCs. Oncol. Rep..

[124] Logozzi M., Angelini D.F., Iessi E., Mizzoni D., Di Raimo R., Federici C., Lugini L., Borsellino G., Gentilucci A., Pierella F. (2017). Increased PSA expression on prostate cancer exosomes in in vitro condition and in cancer patients. Cancer Lett..

[125] Thews O., Gassner B., Kelleher D.K., Schwerd G., Gekle M. (2006). Impact of Extracellular Acidity on the Activity of P-Glycoprotein and the Cytotoxicity of Chemotherapeutic Drugs. Neoplasia.

[126] Kondo A., Yamamoto S., Nakaki R., Shimamura T., Hamakubo T., Sakai J., Kodama T., Yoshida T., Aburatani H., Osawa T. (2017). Extracellular Acidic pH Activates the Sterol Regulatory Element-Binding Protein 2 to Promote Tumor Progression. Cell Rep..

[127] Leon C.G., Locke J.A., Adomat H.H., Etinger S.L., Twiddy A.L., Neumann R.D., Nelson C.C., Guns E.S., Wasan K.M. (2009). Alterations in cholesterol regulation contribute to the production of intratumoral androgens during progression to castration-resistant prostate cancer in a mouse xenograft model. Prostate.

[128] Bersuker K., Hendricks J.M., Li Z., Magtanong L., Ford B., Tang P.H., Roberts M.A., Tong B., Maimone T.J., Zoncu R. (2019). The CoQ Oxidoreductase FSP1 Acts Parallel to GPX4 to Inhibit Ferroptosis. Nat. Cell Biol..

[129] Khutornenko A.A., Roudko V.V., Chernyak B.V., Vartapetian A.B., Chumakov P.M., Evstafieva A.G. (2010). Pyrimidine Biosynthesis Links Mitochondrial Respiration to the p53 Pathway. Proc. Natl. Acad. Sci. USA.

[130] Cheng C., Guo J.Y., Geng F., Wu X., Cheng X., Li Q., Guo D. (2016). Analysis of SCAP N-glycosylation and Trafficking in Human Cells. J. Vis. Exp..

[131] Chiabrando D., Vinchi F., Fiorito V., Mercurio S., Tolosano E. (2014). Heme in pathophysiology: A matter of scavenging, metabolism and trafficking across cell membranes. Front. Pharmacol..

[132] Mullen P.J., Yu R., Longo J., Archer M.C., Penn L.Z. (2016). The interplay between cell signalling and the mevalonate pathway in cancer. Nat. Rev. Cancer.

[133] Gabitova L., Gorin A., Astsaturov I. (2014). Molecular pathways: Sterols and receptor signaling in cancer. Clin. Cancer Res. Off. J. Am. Assoc. Cancer Res..

[134] Zhang Z., Hou X., Shao C., Li J., Cheng J.-X., Kuang S., Ahmad N., Ratliff T., Liu X. (2014). Plk1 Inhibition Enhances the Efficacy of Androgen Signaling Blockade in Castration-Resistant Prostate Cancer. Cancer Res..

[135] Li X., Chen Y.-T., Hu P., Huang W.-C. (2014). Fatostatin displays high antitumor activity in prostate cancer by blocking SREBP-regulated metabolic pathways and androgen receptor signaling. Mol. Cancer Ther..

[136] Csibi A., Lee G., Yoon S.-O., Tong H., Ilter D., Elia I., Fendt S.-M., Roberts T.M., Blenis J. (2014). The mTORC1/S6K1 Pathway Regulates Glutamine Metabolism through the eIF4B-Dependent Control of c-Myc Translation. Curr. Biol..

[137] Karlic H., Varga F., Boffetta P., Hainaut P.B.T. (2019). Mevalonate Pathway. Encyclopedia of Cancer.

[138] Wang K., Gerke T.A., Chen X., Prosperi M. (2019). Association of statin use with risk of Gleason score-specific prostate cancer: A hospital-based cohort study. Cancer Med..

[139] Harshman L.C., Wang X., Nakabayashi M., Xie W., Valenca L., Werner L., Yu Y., Kantoff A.M., Sweeney C.J., Mucci L.A. (2015). Statin Use at the Time of Initiation of Androgen Deprivation Therapy and Time to Progression in Patients With Hormone-Sensitive Prostate Cancer. JAMA Oncol..

[140] Gutt R., Tonlaar N., Kunnavakkam R., Karrison T., Weichselbaum R.R., Liauw S.L. (2010). Statin Use and Risk of Prostate Cancer Recurrence in Men Treated With Radiation Therapy. J. Clin. Oncol..

[141] Hamilton R.J., Banez L.L., Aronson W.J., Terris M., Platz E.A., Kane C.J., Presti J.C., Amling C.L., Freedland S.J. (2010). Statin medication use and the risk of biochemical recurrence after radical prostatectomy. Cancer.

[142] Kumar A., Riviere P., Luterstein E., Nalawade V., Vitzthum L., Sarkar R.R., Bryant A.K., Einck J.P., Mundt A.J., Murphy J.D. (2020). Associations among statins, preventive care, and prostate cancer mortality. Prostate Cancer Prostatic Dis..

[143] Parrales A., Ranjan A., Iyer S., Padhye S., Weir S.J., Roy A., Iwakuma T. (2016). DNAJA1 Controls the Fate of Misfolded Mutant p53 Through the Mevalonate Pathway. Nature.

[144] Murphy C., Deplazes E., Cranfield C.G., Garcia A. (2020). The Role of Structure and Biophysical Properties in the Pleiotropic Effects of Statins. Int. J. Mol. Sci..

[145] Gholkar A., Cheung K., Williams K.J., Lo Y.-C., Hamideh S.A., Nnebe C., Khuu C., Bensinger S.J., Torres J.Z. (2016). Fatostatin Inhibits Cancer Cell Proliferation by Affecting Mitotic Microtubule Spindle Assembly and Cell Division. J. Biol. Chem..

[146] Miyata S., Inoue J., Shimizu M., Sato R. (2015). Xanthohumol Improves Diet-induced Obesity and Fatty Liver by Suppressing Sterol Regulatory Element-binding Protein (SREBP) Activation. J. Biol. Chem..

[147] Tang J.-J., Li J.-G., Qi W., Qiu W.-W., Li P.-S., Li B.-L., Song B.-L. (2011). Inhibition of SREBP by a Small Molecule, Betulin, Improves Hyperlipidemia and Insulin Resistance and Reduces Atherosclerotic Plaques. Cell Metab..

[148] Deeb D., Gao X., Jiang H., Arbab A.S., Dulchavsky S.A., Gautam S.C. (2010). Growth inhibitory and apoptosis-inducing effects of xanthohumol, a prenylated chalone present in hops, in human prostate cancer cells. Anticancer Res..

[149] Venè R., Benelli R., Minghelli S., Astigiano S., Tosetti F., Ferrari N. (2012). Xanthohumol impairs human prostate cancer cell growth and invasion and diminishes the incidence and progression of advanced tumors in TRAMP mice. Mol. Med..

[150] Reiner T., Parrondo R., de Las Pozas A., Palenzuela D., Perez-Stable C. (2013). Betulinic acid selectively increases protein degradation and enhances prostate cancer-specific apoptosis: Possible role for inhibition of deubiquitinase activity. PLoS ONE.

[151] Cheng C., Geng F., Cheng X., Guo D. (2018). Lipid metabolism reprogramming and its potential targets in cancer. Cancer Commun..

[152] Pandyra A.A., Mullen P.J., Goard C.A., Ericson E., Sharma P., Kalkat M., Yu R., Pong J.T., Brown K., Hart T. (2015). Genome-wide RNAi analysis reveals that simultaneous inhibition of specific mevalonate pathway genes potentiates tumor cell death. Oncotarget.

[153] Zhu M.-L., Horbinski C.M., Garzotto M., Qian D.Z., Beer T.M., Kyprianou N. (2010). Tubulin-targeting chemotherapy impairs androgen receptor activity in prostate cancer. Cancer Res..

[154] Meor Anuar Shuhaili M.F.R., Samsudin I.N., Stanslas J., Hasan S., Thambiah S.C. (2017). Effects of Different Types of Statins on Lipid Profile: A Perspective on Asians. Int. J. Endocrinol. Metab..

[155] Menter D.G., Ramsauer V.P., Harirforoosh S., Chakraborty K., Yang P., Hsi L., Newman R.A., Krishnan K. (2011). Differential Effects of Pravastatin and Simvastatin on the Growth of Tumor Cells from Different Organ Sites. PLoS ONE.

[156] Caro-Maldonado A., Camacho L., Zabala-Letona A., Torrano V., Fernández-Ruiz S., Zamacola-Bascaran K., Arreal L., Valcárcel-Jiménez L., Martín-Martín N., Flores J.M. (2017). Low-dose statin treatment increases prostate cancer aggressiveness. Oncotarget.

[157] Murtola T.J., Syvälä H., Tolonen T., Helminen M., Riikonen J., Koskimäki J., Pakarainen T., Kaipia A., Isotalo T., Kujala P. (2018). Atorvastatin Versus Placebo for Prostate Cancer Before Radical Prostatectomy-A Randomized, Double-blind, Placebo-controlled Clinical Trial. Eur. Urol..

[158] Longo J., Hamilton R.J., Masoomian M., Khurram N., Branchard E., Mullen P.J., Elbaz M., Hersey K., Chadwick D., Ghai S. (2020). A pilot window-of-opportunity study of preoperative fluvastatin in localized prostate cancer. Prostate Cancer Prostatic Dis..

[159] Rajora M.A., Zheng G. (2016). Targeting SR-BI for Cancer Diagnostics, Imaging and Therapy. Front. Pharmacol..

[160] Sarhadi S., Ganjali S., Pirro M., Sahebkar A. (2019). The role of high-density lipoproteins in antitumor drug delivery. IUBMB Life.

[161] McMahon K.M., Plebanek M.P., Thaxton C.S. (2016). Properties of Native High-Density Lipoproteins Inspire Synthesis of Actively Targeted In Vivo siRNA Delivery Vehicles. Adv. Funct. Mater..

[162] Sabnis S., Sabnis N.A., Raut S., Lacko A.G. (2017). Superparamagnetic reconstituted high-density lipoprotein nanocarriers for magnetically guided drug delivery. Int. J. Nanomed..

[163] Bray F., Ferlay J., Soerjomataram I., Siegel R.L., Torre L.A., Jemal A. (2018). Global cancer statistics 2018: GLOBOCAN estimates of incidence and mortality worldwide for 36 cancers in 185 countries. CA Cancer J. Clin..

[164] Maitland N.J. (2017). Getting closer to prostate cancer in patients—What scientists should want from clinicians. J. Cancer Metastasis Treat..

[165] Latonen L., Afyounian E., Jylhä A., Nättinen J., Aapola U., Annala M., Kivinummi K.K., Tammela T.T.L., Beuerman R.W., Uusitalo H. (2018). Integrative proteomics in prostate cancer uncovers robustness against genomic and transcriptomic aberrations during disease progression. Nat. Commun..

